# Sickness Insurance

**Published:** 1916-02

**Authors:** Ira W. Bullard

**Affiliations:** Opelika, Ala.


### SICKNESS INSURANCE.

### A Preventive of the Neglect of Sick Individuals, of Charity Practice and of Unpaid Doctor's Bills

By Ira W. Bullard, M. D., Opelika, Ala.

Industrial hygiene and the health of the people whose daily wage depends so largely on their physical efficiency, have come to constitute one of our most serious industrial problems.

The magnitude of the problem can hardly be comprehended. There are approximately 30,000,000 wage earners in the United States. Each one of these loses on an average about nine days each year on account of sickness. Estimating the daily wages of each of these individuals to average $2.00 and the cost of medical service for each day to average $2.50, the annual loss would amount to around $1,215,000,00. Now, with this loss of $40.50 each year for each wage earner of the family and the same amount of sickness for each non-wage earner of the family with its attendant expense of $22.50 each, it soon becomes apparent that the probable amount that must be paid for the maintenance of health, based upon the average of this immense number of persons, becomes an item to be reckoned with and in many instances a cause of serious embarassment.

Seldom do we consider the economic reasons for trying to keep well, yet, they are among the most vital reasons of all. A man will warn his son to avoid carelessness and foolishness that might make him sick or handicap him for life by saying, "If you get sick you will have to go to bed, take disagreeable medicines, be kept from your pleasures—and you might die." He may warn the youths thus and yet not say a word about the inability of the family to bear the expense of sickness.

Actuaries have made it easy for us to state, with some approach at accuracy, the actual money value of health, the loss to the community through sickness from loss of earning capacity and the expense that must be paid in cash. They have also shown us the vast public expense from child mortality. Not only is all sickness expensive but sickness interrupts the well thought out plans of individuals, families and communities and history records instances in which sickness has defeated armies and destroyed nations.

The figures are fairly correct as to the ultimate cost and the loss to the community from sickness. Such loss is largely in the cutting off of possible future gains rather than the loss of actual cash, except the sickness expense and the public is thus slow to believe in the figures. However, it has been definitely worked out that the pecuniary worth, potential and prospective, of an individual at the different ages is as follows:

| | |
|---|---|
| At Birth . . . . . . . . | $ 90.00 |
| At Age 5 . . . . . . . | 950.00 |
| At Age 10 . . . . . . . | 2000.00 |
| At Age 20 . . . . . . . | 4000.00 |
| At Age 30 . . . . . . . | 4100.00 |
| At Age 40 . . . . . . . | 2900.00 |
| At Age 50 . . . . . . . | 1700.00 |

The value of the life is figured on the possible earning power through the years that are promised for the individual.

At present medical practice in the United States is operating on a most uneconomical and unsocial basis. Many men with an average income cannot afford to pay the charges for the average sickness expectancy to the amount shown above. Furthermore physicians are practicing medicine against heavy odds because of the inability of the average man to pay this high rate of charge that the physician is compelled to make in order to strike a balance and assure himself a living out of the work for those who cannot pay as well as for those who can. Even at that, it is estimated that the average income of the physicians of the United States is $750.00 per year.

The result of the present day plan is, that the physician, unable to collect his bill for services rendered in the past stops his service to this particular individual or family, unless the said individual publicly proclaims himself an object of charity, and refuses to attend him further. He thereby loses all probability of collecting this bill and the individual patient, perhaps having had several such experiences or perchance this is his first, at any rate develops a feeling of resentment towards his former physician, which is often reciprocated on the physician's part, and two men become estranged, hard feelings are engendered, the one loses the amount of his bill, the other his self-respect in not being able to pay the bill. Other members sick in the family are perhaps neglected because of the additional bill that will have to be made with some other physician and many die every year that could be saved. Untold numbers become totally disabled and unable to work, their wives become invalids from improper care during confinement and thousands of their children die during the first year of life from lack of intelligent care, all through the fear of the expense or the actual inability to pay the high cost of competent medical service.

The waste from disability and death due to preventable diseases is so tremendous that estimates mean nothing to the average mind. The sufferings due to these causes alone should be sufficient argument for some radical change from the present one-sided and ineffective policy and place adequate medical service in the reach of all.

How is such a change to be brought about? With our present light we can descern but one satisfactory plan. That plan is sickness insurance. What is sickness insurance? Insurance of any kind, in its last analysis, is that method by which a group of persons, each liable to some danger or some loss, the incidence of which cannot be foreseen, can distribute the cost of such loss, where it occurs to any one of them, over the whole group and thus lighten the burden of its most serious economic effects.

This division of the costs would greatly reduce the amount now spent by any one individual and in addition distribute the payment over such a long period of time that any one could meet the expense without lowering his present standard of living or working a hardship upon himself or his family, and furthermore this expense would be met in a business-like manner and leave no "taint of charity." By this plan more than by any other will the economic independence of the individual be established and also the income of the physician be stabilized and made certain.

Such sickness insurance, in the course of time will unquestionably become a governmental or federal system as it already has in Great Britain, Germany, Switzerland, Austria and in fact practically all of the European nations and it is a noteworthy fact that no nation that enacted laws on this subject has ever repealed them but rather they are being continually extended.

Far-sighted leaders of the medical profession in America are looking forward to the adaption of such a plan of medical service, as witness the remarkable report of The Judicial Council of the American Medical Association presented last June at the San Francisco session of that body. A bill establishing sickness insurance will be introduced into the New York legislature in 1916 and there are already thirty-three states in the Union that have adopted some form of workman's compensation laws. It is but a question of time until these will be extended to include sickness insurance as well as insurance against injury while at work.

The insurance act adopted by Great Britain a few years since is the most comprehensive yet extant. The point of greatest difficulty both in Great Britain and in Germany has been the organization of the medical service. In both countries there were severe conflicts between the governments and the medical societies. However, nine-tenths of the physicians of these countries at this writing are on the so-called insurance panels. Each physician is paid on a basis of so much per year for each person on his panel, the payment being made monthly, quarterly, semi-annually or annually. The result in the case of the physicians has been that their average incomes has been increased from $750.00 to $2000 per year and in the case of the people the results have been no less advantageous. Previous to the passage of this law of sickness insurance, millions of people had either no medical attendance at all or received it only in great emergencies and then under severe strain because of the expense, while now medical service is made accessible to the whole population because of the act having vastly extended the reach of medical practice.

In the German and English sickness insurance systems, voluntary organizations or individuals make their own insurance arrangements with medical men under the supervision of the State Insurance Department. Such groups also make arrangements for their own medical service, under the insurance system with dispensaries and hospitals. Under both the British and German systems all persons having less than a specified minimum income must join some group unde the compulsory insurance act while presons having a higher grade of income are allowed to take sickness insurance as so-called "voluntary insurers," and the formation of these voluntary mutual insurance groups is encouraged by the government in every possible way. The strongest encouragement to the individual is the possibility of securing high-grade medical service at a lower cost than would be possible without co-operation in such groups. In the United States there is a growing recognition of the difficulty with which the great mass of the middle classes, professional men, merchants, farmers, salaried men can secure the same grade of medical service open to the rich, who are not mindful of the high cost, and to the very poor as charity patients. By making up such volunteer insurance groups, the same grade of service can be financed for the great mass of the people and by co-operation on the part of the physicians, using the principle of medical team work and a joint diagnostic and hospital equipment, the service can be made very efficient from the medical standpoint.

To test out methods of medical service on the team work plan through such voluntary insurance groups is very important and a duty to which the medical profession and the public at large should diligently apply themselves. Either the equipment afforded by an existing hospital, which we have not and probably never will have until it is developed along this line or provided by a group of doctors associating themselves together for that purpose, will unquestionably prove successful and to a high degree satisfactory both to the physicians concerned and to the public.

This is a new development of medical practice in the South, but one that is far advanced in the East and middle West and to which my attention was forcibly drawn upon a recent visit to Chicago. For several years large corporations here in the South have acknowledged the vast advantages of sickness insurance among their employees from the purely business standpoint of increased efficiency and have organized their entire employee list into a sickness insurance group of which each individual pays into the insurance fund each month a certain amount depending upon the number of individuals in his family. Such monthly payments are in the same sense insurance premiums as are the sums paid annually or semi-annually upon life insurance policies and they insure or guarantee the payor adequate medical service at no additional cost in the event of sickness as the life insurance premiums insure the payment of a certain sum of money in the event of death.

If corporations thus find it a wise and conservative business proposition to insure their employees adequate medical service without the severe economic strain attendant upon the usual procuring of such service, why should not the public at large find it equally wise and conservative?

We believe that they would and it appears to us as a good business proposition. If we have before us at the beginning of the year the very great probability that each member of our family, in order to maintain his health and efficiency at the highest possible level, will require the services of a physician upon at least nine different occasions during the year at an average cost of $2.00 for each occasion and the possibility that some member of the family may require a physician ninety and nine times instead of nine, is it not good business to pay for these nine times at so much a month and thus guarantee ourselves adequate medical services for the number of occasions that statistics show we will in all probability require them but in addition guarantee ourselves adequate medical services for the ninety and nine times that we might require them and without any additional cost.

But the greatest advantage of sickness insurance is not upon these nine occasions when we are sufficiently ill to require medical services, but upon those numerous occasions when preventive medicine, an ever broadening field of medical practice, could exercise its powerful influence in wholly preventing or certainly allaying disease but which, under the present conditions of medical practice, the vast majority of people do not take advantage of. The most commonly expressed reason for this that I have heard is, that they do not feel justified in paying the high cost of medical service for conditions that they think are of minor importance.

Physicians realize that many of these conditions that the average person considers of minor importance are in truth the outcroppings of serious conditions that are finally brought to the attention of the physician only after they have developed a degree of pathology that makes relief surely less certain than would have been the case with earlier attention, as witness Brights Disease in men, cancer in women and tuberculosis in childhood and adolescence.

The amount of service performed at present by the average physician along the lines of preventive medicine is but small indeed compared to what it should be and the reasons are that the public does not appreciate the importance of preventive medicine on the one hand and on the other do not feel justified in paying the cost of medical service for the care of conditions that they do not think require such service. The result is the large percentage of preventable sickness and death that every year pushes up the mortality records of every community.

The solving of this problem is well worth while and we believe that its solution is some form of sickness insurance of which there are several well-adapted plans that have already been worked out and proven highly successful.

